# A Further Comment on ‘Large-Scale Cognitive GWAS Meta-Analysis Reveals Tissue-Specific Neural Expression and Potential Nootropic Drug Targets’ by Lam et al.

**DOI:** 10.1017/thg.2018.55

**Published:** 2018-10-08

**Authors:** W. D. Hill

**Affiliations:** 1 Centre for Cognitive Ageing and Cognitive Epidemiology, University of Edinburgh, Edinburgh, UK; 2 Department of Psychology, University of Edinburgh, Edinburgh, UK

**Keywords:** GWAS, general cognitive ability, nootropics, gene expression, neurodevelopment, synapse, calcium channel, potassium channel, cerebellum

## Abstract

Lam et al. (2018) respond to a commentary of their paper entitled ‘Large-Scale Cognitive GWAS Meta-Analysis Reveals Tissue-Specific Neural Expression and Potential Nootropic Drug Targets’ Lam et al. (2017). While Lam et al. (2018) have now provided the recommended quality control metrics for their paper, problems remain. Specifically, Lam et al. (2018) do not dispute that the results of their multi-trait analysis of genome-wide association study (MTAG) analysis has produced a phenotype with a genetic correlation of one with three measures of education, but do claim the associations found are specific to the trait of cognitive ability. In this brief paper, it is empirically demonstrated that the phenotype derived by Lam et al. (2017) is more genetically similar to education than cognitive ability. In addition, it is shown that of the genome-wide significant loci identified by Lam et al. (2017) are loci that are associated with education rather than with cognitive ability.

Lam et al. (2018) assert that their use of multi-trait analysis of genome-wide association study (GWAS) (MTAG; Turley et al., 2018) resulted in the generation of associations that were specific to the trait of cognitive ability. They offer four pieces of supporting evidence based on the genetic correlations derived using linkage disequilibrium score (LDSC) regression (Bulik-Sullivan et al., 2015) found in Table S14 of Lam et al. (2018).

First, Lam et al. (2018) state that ‘our Table S14 demonstrates the set of genetic correlations for the MTAG data differ from those of educational attainment (from the Okbay et al. (2016) data set) in many cases by an absolute value (for *r*_g_) of 0.10 or greater’. This characterization of the genetic correlations in Table S14 of Lam et al. (2017) differing between the MTAG phenotype and education is misleading. This can be shown empirically by first extracting the genetic correlations from Table S14 from Lam et al. (2017). Next, in order to have confidence in the accuracy of the point estimates of the genetic correlations, we extract genetic correlations that were significant for at least one of the cognitive phenotypes (Education, the MTAG phenotype, or a GWAS composed of solely cognitive measures labeled as cognitive ability). This leaves 61 traits that show a nominally significant genetic correlation with one of the cognitive traits used by Lam et al. (2017). Next, we test for significant differences between the genetic correlations derived using education and these 61 traits, with the genetic correlations derived using the MTAG phenotype and the same 61 traits.

The results of this analysis can be seen in Figure 1, and show the only six instances out of 61 traits where there was a significantly different genetic correlation between the MTAG phenotype and education. Crucially, in each of these six instances, the genetic correlations produced using the MTAG phenotype are also significantly different from those produced using cognitive ability. This indicates that in most instances there are no differences between education and the MTAG phenotype; however, in the instances where the MTAG phenotype deviates from education, it is also significantly different from cognitive ability. Thus, the conclusion of Lam et al. (2018) that their MTAG phenotype is more similar to cognitive ability, because there are instances where the point estimates between education and the MTAG phenotype differ, is empirically falsified.

**FIGURE 1:**
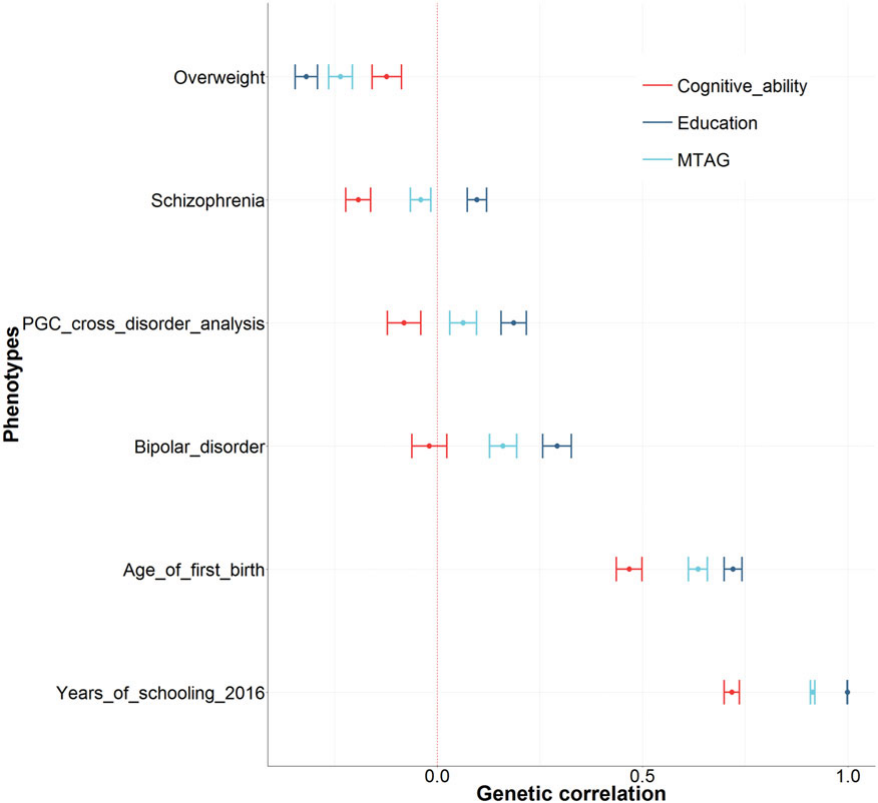
An empirical comparison between the genetic correlations produced using the three cognitive phenotypes from Lam et al. (2017). First, genetic correlations were selected when there was at least one nominally significant genetic correlation with any one of the three cognitive phenotypes used in Lam et al. Second, we show that for those traits, there were only six instances where the MTAG phenotype was significantly different from the education phenotype. However, in each of these six instances, the MTAG phenotype was also significantly different from the cognitive ability phenotype. Each point represents a genetic correlation between one of the three cognitive phenotypes (red = cognitive ability, dark blue = education, and light blue = the MTAG phenotype described as trait specific to cognitive ability by Lam et al. (2017)) and the traits presented on the *y*-axis. The dotted red line indicates a genetic correlation of zero. Error bars represent ±1 standard error as derived using LDSC regression.

Second, in their response, Lam et al. (2018) draw special attention to the genetic correlations between childhood IQ and their MTAG phenotype, writing that it is ‘virtually identical’ to that produced using cognitive ability and childhood IQ, and that there is a ‘notably smaller’ genetic correlation between education and childhood IQ. Infant head circumference is also mentioned as producing a genetic correlation with a point estimate that is greater when using the MTAG phenotype rather than using either education or cognitive ability. While in their rebuttal, Lam et al. (2018) provide point estimates for these genetic correlations, no measure is given for their standard error, and no estimate is given for the magnitude of the difference between these genetic correlations. These standard errors can again be found in Table S14 of Lam et al. (2017) and, when examined, show that the genetic correlations derived using childhood IQ and each of the three cognitive phenotypes used by Lam et al. (2017) show no significant difference between the education-childhood IQ and the MTAG phenotype-childhood IQ genetic correlations (*p* = .19), the MTAG phenotype-childhood IQ and cognitive ability-childhood IQ genetic correlations (*p* = .90), nor between the education-childhood IQ and cognitive ability-childhood IQ genetic correlations (*p* = .14). This same trend is again seen when examining infant head circumference. In that case, the following results are found when comparing the same three pairs of genetic correlations: education and the MTAG phenotype (*p* = .65), the MTAG phenotype and cognitive ability (*p* = .78), and education and cognitive ability (*p* = .90). This indicates that far from being counter examples to the arguments put forward by Hill (2018), these phenotypes, selected by Lam et al. (2018) as exemplars indicating the success of their MTAG approach, show no evidence for their claim that their MTAG phenotype is more similar to cognitive ability than education.

The third piece of evidence Lam et al. (2018) use to show that their MTAG analysis is more similar to cognitive ability than to education, is to claim the magnitude of the genetic correlations with education were reported inaccurately by Hill (2018). In their rebuttal, Lam et al. (2018) write that ‘Hill (2018) elides the fact that the calculation method employed by LD score regression is known to sometimes produce values for *r*_g_ >1, if the variables are so highly similar as to be self-same (Walters, 2016)’. However, Hill (2018) states in his Table 1, Figure 1, and in the publically available scripts used by Hill (2018) to perform his analyses that genetic correlations of greater than 1 are being treated as 1. In addition, the same reference (Walters, 2016) is used in the rebuttal by Lam et al. (2018) to state this is a known issue of LDSC regression, as is used by Hill (2018) to justify the decision to report genetic correlations of greater than 1 as 1. The relevant text from Walters (2016) on how to interpret genetic correlations of greater than 1 is included verbatim here: ‘Thus, far our interpretation has been that as long as artifacts or instability have been ruled out then this result indicates *r*_g_ approx. = 1’.

It should be noted that if one derives a genetic correlation using LDSC regression using the same data set correlated against itself, a genetic correlation of exactly 1 is produced. Genetic correlations of greater than one appear when two highly similar measures of the same phenotype (e.g., years of education vs. college degree coded as a binary variable) are used. Importantly, as shown in the rebuttal by Lam et al. (2018), a genetic correlation derived using the MTAG phenotype and the GWAS derived only using cognitive tests used in the construction of the MTAG phenotype did not produce this effect. Furthermore, by following the advice of Walters (2016) in reporting genetic correlations of greater than 1 as 1, we avoid the issue of a variable being more similar to a second variable than it is with itself. Together, this shows that the MTAG phenotype derived by Lam et al. (2017) does indeed show no significant difference in the genetic correlations derived with three measures of education compared with genetic correlations between a GWAS of education and the same three measures of education as shown in Hill (2018), Figure 1.

It should be noted that the magnitude of the genetic correlations between the MTAG phenotype and three measures of education are 0.98 (*SE* = 0.03), 0.97 (*SE* = 0.02), and 0.97 (*SE* = 0.03), none of which are significantly different to 1. Figure 1 of Hill (2018) also shows that there was a significant difference between the genetic correlations derived using the MTAG phenotype and those derived using cognitive ability, showing that the genetic architecture of these two traits differ significantly with how they overlap with four measures of education (College completion *p* = 2.29 × 10^−7^, Years of schooling *p* = 8.14 × 10^−8^, Years of schooling 2013 *p* = 9.15 × 10^−8^, Years of schooling 2016 *p* = 2.70 × 10^−25^). Neither the fact that the MTAG phenotype shows a genetic correlation near 1 with three measures of education (just the genetic correlations between education and different measures of education were contested) nor the significantly different genetic correlations between the MTAG phenotype and cognitive ability are disputed in the rebuttal by Lam et al. (2018). Again, then, it appears that the Lam et al. (2017) MTAG phenotype is more similar to education than to cognitive ability.

Fourth, Lam et al. (2018) also believe that the results of their genetic correlations with schizophrenia and bipolar disorder highlight a biologically meaningful set of relationships rather than being the result of their MTAG phenotype having a genetic correlation at unity with education as shown in Figure 1 of Hill (2018). Lam et al. (2018) also state that the ‘overall pattern of genetic correlations’ between the three cognitive phenotypes used in Lam et al. (2017) — education, the MTAG-intelligence phenotype, and a GWAS composed solely of tests of cognitive ability — is ‘highly similar’. However, attention was called to the results of bipolar disorder and of schizophrenia by Hill (2018) precisely because genetic correlations between education with bipolar disorder and schizophrenia are positive (Bulik-Sullivan et al., 2015; Hill et al., 2015; Okbay et al., 2016), whereas for cognitive ability they are negative for schizophrenia and near zero for bipolar disorder (Hagenaars et al., 2016; Hill et al., 2015). This separation provides the ability to examine whether the associations produced by Lam et al. (2017) are indeed trait-specific to cognitive ability as claimed, or are in fact closer to education as shown by Hill (2018).

This too can be investigated by using the data provided by Lam et al. (2017); again, following the extraction of genetic correlations that were nominally significant with at least one of the cognitive traits found in Lam et al. (2017), we are left with genetic correlations between education, cognitive ability, and the MTAG derived phenotype, and 61 traits (Figure 2). As can be seen in Figure 2, there is more similarity between the point estimates of the education and the MTAG phenotype than there is between the point estimates of a GWAS composed solely of tests of cognitive ability and the MTAG phenotype.

**FIGURE 2:**
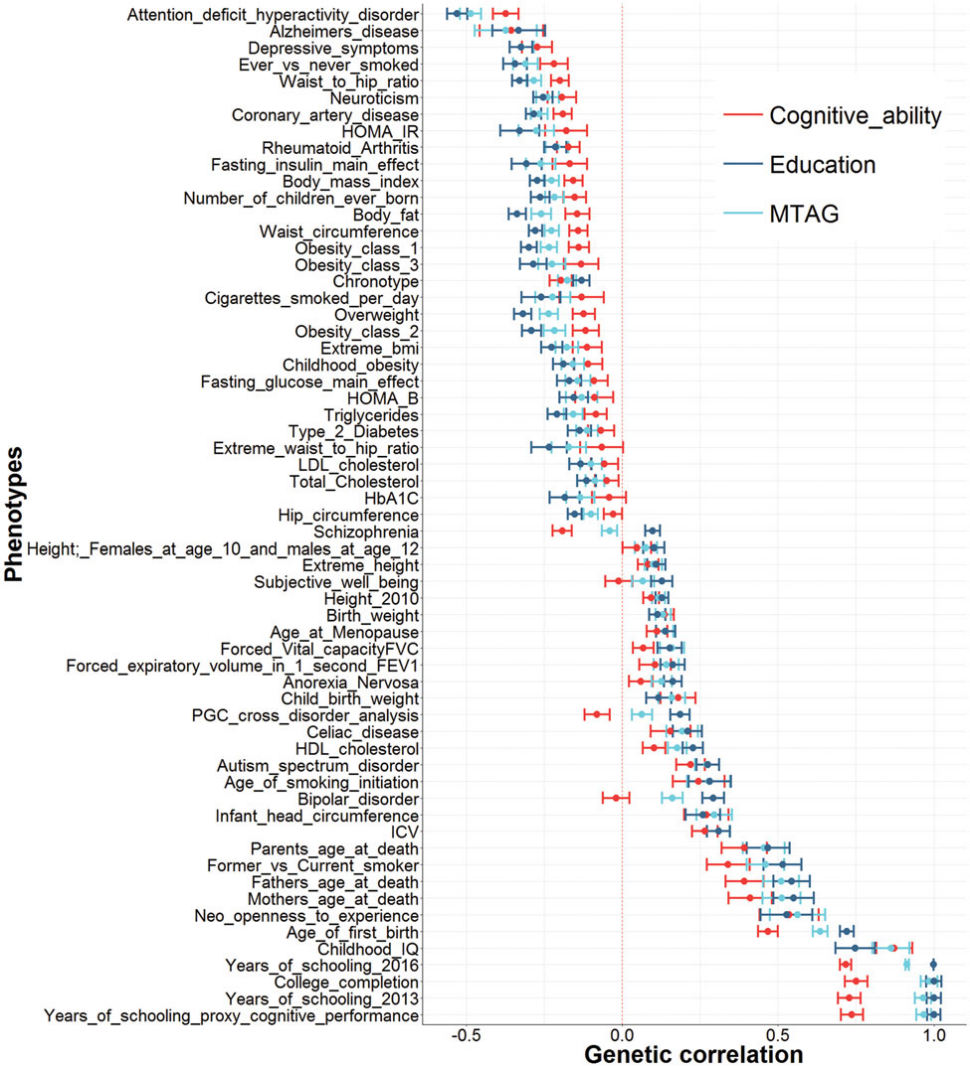
Genetic correlations from Lam et al. (2017) where at least one of the cognitive traits showed a nominally significant genetic correlation with the traits presented on the *y*-axis. Each point represents a genetic correlation between one of the three cognitive phenotypes (red = cognitive ability, dark blue = education, and light blue = the MTAG phenotype described as trait specific to cognitive ability by Lam et al. (2017)) and the traits presented on the *y*-axis. The dotted red line indicates a genetic correlation of zero. Error bars represent ±1 standard error as derived using LDSC regression.

Next, from these 61 traits, we extract instances of where the genetic correlations are significantly different between education and cognitive ability (Figure 3). This leaves 27 traits. As can be seen in Figure 3, the point estimates of these genetic correlations are more similar between education and the MTAG phenotype than between the MTAG phenotype and cognitive ability.

**FIGURE 3:**
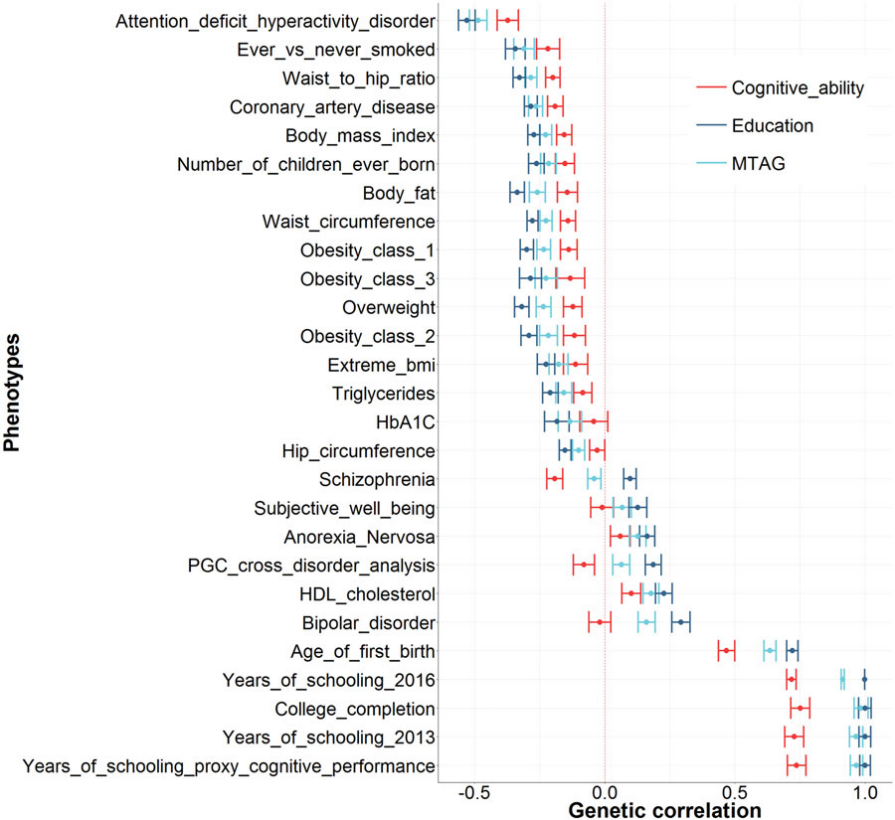
A subset of the traits showed in Figure 2. This shows the 27 traits that are significantly different between education and cognitive ability. Note that in every instance the point estimate of the genetic correlation for the MTAG phenotype is closer to the point estimate of education than it is to cognitive ability. Each point represents a genetic correlation between one of the three cognitive phenotypes (red = cognitive ability, dark blue = education, and light blue = the MTAG phenotype described as trait specific to cognitive ability by Lam et al. (2017)) and the traits presented on the *y*-axis. The dotted red line indicates a genetic correlation of zero. Error bars represent ±1 standard error as derived using LDSC regression.

We then test to determine whether there is a significant difference between the genetic correlations derived using education and those derived using the MTAG phenotype. In Figure 4, we see these results show that 21 out of these 27 traits show no evidence of the MTAG phenotype being significantly different from education and, as stated above, each of the six phenotypes that was significantly different from education was also significantly different from cognitive ability (Figure 1). Of note are the three phenotypes highlighted in red, showing that the MTAG phenotype has a genetic correlation of 1 with each of these three measures of education.

**FIGURE 4:**
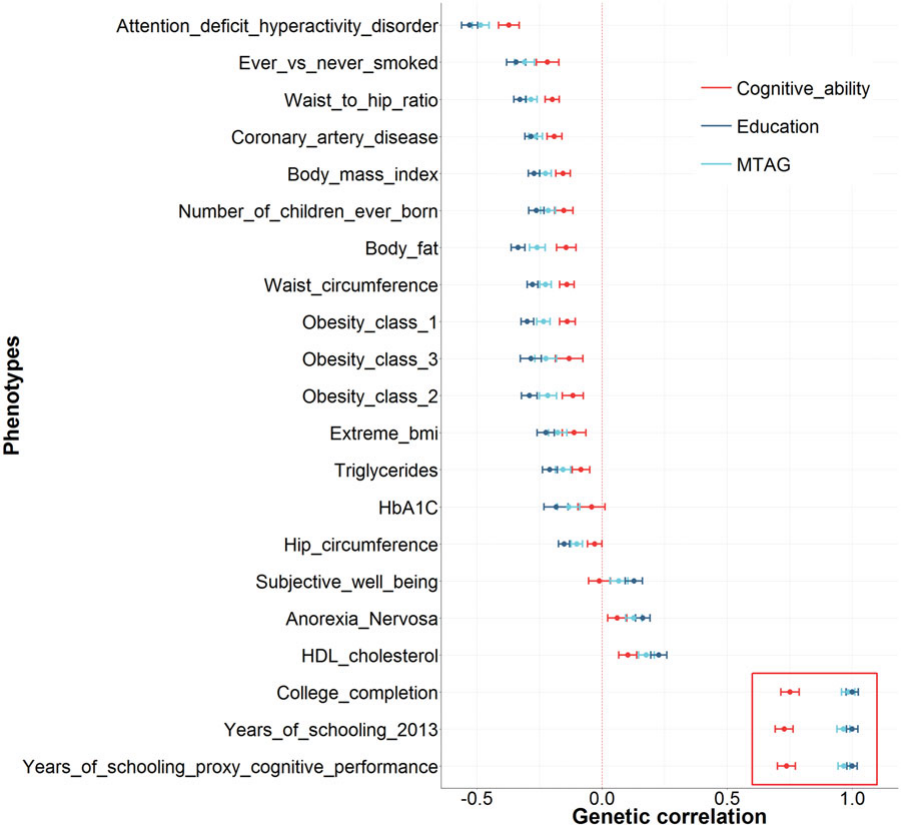
A subset of the traits showed in Figure 3. This shows the 21 traits that show no significant difference between education and the MTAG phenotype. Note that in every instance where a significant difference was found between MTAG phenotype and education, a significant difference was also found between the MTAG phenotype and cognitive ability as shown in Figure 1. Each point represents a genetic correlation between one of the three cognitive phenotypes (red = cognitive ability, dark blue = education, and light blue = the MTAG phenotype described as trait specific to cognitive ability by Lam et al. (2017)) and the traits presented on the *y*-axis. The dotted red line indicates a genetic correlation of zero. Error bars represent ±1 standard error as derived using LDSC regression. The red box highlights the genetic correlations with three measures of education and shows that the MTAG phenotype has a genetic correlation of 1 with each of them.

The above series of analyses invalidates the claims of Lam et al. (2017) that their MTAG phenotype is trait specific to cognitive ability and separate from education. As shown, in instances where the genetic correlations produced using education and cognitive ability differ (Figure 3) the MTAG phenotype shows no significant differences between those derived using education in 21 out of 27 cases. Where such differences are evident, the MTAG phenotype produces genetic correlations that are also significantly different from those produced using cognitive ability (Figure 1). Thus, contrary to the claims of Lam et al. (2017), that their MTAG phenotype is trait-specific to cognitive ability, it is empirically shown here that their MTAG phenotype is more similar to education and significantly different from established measures of cognitive ability. Lam et al. (2018) also state ‘our leave-one-out analyses (Figure 3 in Lam et al. ((2017)) demonstrate that prediction of held-out samples, phenotyped for cognitive ability, are better for MTAG than for either GWAS_COG_ or GWAS_EDU_ alone. This finding supports our interpretation that MTAG is boosting polygenic signal for cognition, and does not support the conclusion of Hill (2018) that the MTAG polygenic signal is ‘indistinguishable from that of education’’. The phrase ‘indistinguishable from that of education’ is attributed to Hill (2018) but it is not found in the manuscript of Hill (2018). However, the point raised, that a polygenic risk score derived using the MTAG phenotype explains more variance in cognitive ability than a polygenic risk score derived using education does, is discussed below.

Polygenic risk score analysis involves deriving an individual level predictor using the summary GWAS data from one sample, and the phenotypic and genotypic data from a second, independent, data set. This can be done within the same trait, such as by using the summary GWAS statistics from one data set on education to predict phenotypic levels of education in an independent data set. Alternatively, cross-trait polygenic risk scores can be used, whereby using the summary GWAS data on, for example, cognitive ability is used to predict phenotypic variance for education. Importantly, cross-trait polygenic risk scores can only predict phenotypic variance in instances where there is a non-zero genetic correlation between the two traits. However, unlike genetic correlations, polygenic risk scores do not indicate the degree to which genetic effects are shared, only that there is a non-zero shared genetic effect across the two traits. In order to empirically quantify the proportion of genetic effects that are shared across traits, genetic correlations are needed. As shown above, by using genetic correlations, the MTAG phenotype more closely resembles education than cognitive ability.

It should be noted that Lam et al. (2018) have now included the maxFDR rate, as suggested by the authors of MTAG (Turley et al., 2018). The maxFDR was 0.0068, indicating that between 0 and 1 of these loci was a false positive. Lam et al. (2018) also claim that ‘the top results (genome-wide significant loci) emerging from MTAG show notable differences from those emerging from GWAS_EDU_, but are almost a complete superset of those emerging from GWAS_COG_’. Larger data sets are now available to test this assertion. Using publically GWAS data from 766,345 individuals who provided information on whether or not they had a college or university level degree (Lee et al, 2018) and the largest publically available data set on cognitive ability (*n* = 168,033) that does not also include measures of education (Davies et al., 2018), we examine the claims of Lam et al. (2018). First, we extract the 82 independent lead SNPs found in Lam et al.’s (2017) Table S1. We find that of the 82 genome-wide significant SNPs reported by Lam et al. (2017), 81 are present in the (Lee et al., 2018) education data set where 65 are genome-wide significant. For the cognitive ability data set of Davies et al. (2018), we find that 82 of the genome wide SNPs from Lam et al. (2017) are present. However, only 29 of these are genome-wide significant for cognitive ability. This result demonstrates that the genome-wide significant SNPs of the MTAG phenotype derived by Lam et al. (2017) show a greater overlap with education than with cognitive ability. Table S1 shows the association statistics for each of the 82 genome-wide significant lead SNPs from Lam et al. (2017) as well as the association statistics for the same SNP for education (Lee et al., 2018), and for cognitive ability (Davies et al., 2018).

As shown above, the phenotype derived by Lam et al. (2017) using MTAG shows a genetic correlation of 1 with three measures of education (Figure 1 of Hill (2018) and Figure 4 in the current paper), an issue that is not contested in the response by Lam et al. (2018). Furthermore, where the pattern of genetic correlations differs between education and cognitive ability (27 traits), the pattern of genetic correlations derived using their MTAG phenotype shows no significant differences with those derived using education (21 traits). Where such differences are found between the genetic correlations produced using education and the MTAG phenotype, the MTAG phenotype produces genetic correlations that are significantly different from those produced using cognitive ability (six traits). Finally, well-powered GWAS are showing that the loci identified by Lam et al. (2017) have more in common with education than with cognitive ability. For these empirical reasons, it is difficult to understand the position of Lam et al. (2017), who claim this phenotype, derived using MTAG, has produced associations that are specific to the trait of cognitive ability; rather, it seems much more likely that these associations have more relevance to education than to cognitive ability.

## Supplementary material

For supplementary material accompanying this paper visit https://doi.org/10.1017/thg.2018.55. click here to view supplementary material
